# Improved Dipper-Throated Optimization for Forecasting Metamaterial Design Bandwidth for Engineering Applications

**DOI:** 10.3390/biomimetics8020241

**Published:** 2023-06-07

**Authors:** Amal H. Alharbi, Abdelaziz A. Abdelhamid, Abdelhameed Ibrahim, S. K. Towfek, Nima Khodadadi, Laith Abualigah, Doaa Sami Khafaga, Ayman EM Ahmed

**Affiliations:** 1 Department of Computer Sciences, College of Computer and Information Sciences, Princess Nourah bint Abdulrahman University, P.O. Box 84428, Riyadh 11671, Saudi Arabia; ahalharbi@pnu.edu.sa (A.H.A.); dskhafga@pnu.edu.sa (D.S.K.); 2 Department of Computer Science, College of Computing and Information Technology, Shaqra University, Shaqra 11961, Saudi Arabia; abdelaziz@su.edu.sa; 3 Department of Computer Science, Faculty of Computer and Information Sciences, Ain Shams University, Cairo 11566, Egypt; 4 Computer Engineering and Control Systems Department, Faculty of Engineering, Mansoura University, Mansoura 35516, Egypt; 5 Computer Science and Intelligent Systems Research Center, Blacksburg, VA 24060, USA; sktowfek@jcsis.org; 6 Department of Communications and Electronics, Delta Higher Institute of Engineering and Technology, Mansoura 35111, Egypt; 7 Department of Civil and Architectural Engineering, University of Miami, Coral Gables, FL 33146, USA; 8 Hourani Center for Applied Scientific Research, Al-Ahliyya Amman University, Amman 19328, Jordan; aligah.2020@gmail.com; 9 MEU Research Unit, Middle East University, Amman 11831, Jordan; 10 School of Computer Sciences, Universiti Sains Malaysia, Gelugor 1800, Penang, Malaysia; 11 Computer Science Department, Prince Hussein Bin Abdullah Faculty for Information Technology, Al al-Bayt University, Mafraq 25113, Jordan; 12 Faculty of Engineering, King Salman International University, El-Tor 11341, Egypt; ayman.ahmed@alumni.carleton.ca

**Keywords:** forecasting wind power, Al-Biruni earth radius, metaheuristic algorithm, artificial intelligence

## Abstract

Metamaterials have unique physical properties. They are made of several elements and are structured in repeating patterns at a smaller wavelength than the phenomena they affect. Metamaterials’ exact structure, geometry, size, orientation, and arrangement allow them to manipulate electromagnetic waves by blocking, absorbing, amplifying, or bending them to achieve benefits not possible with ordinary materials. Microwave invisibility cloaks, invisible submarines, revolutionary electronics, microwave components, filters, and antennas with a negative refractive index utilize metamaterials. This paper proposed an improved dipper throated-based ant colony optimization (DTACO) algorithm for forecasting the bandwidth of the metamaterial antenna. The first scenario in the tests covered the feature selection capabilities of the proposed binary DTACO algorithm for the dataset that was being evaluated, and the second scenario illustrated the algorithm’s regression skills. Both scenarios are part of the studies. The state-of-the-art algorithms of DTO, ACO, particle swarm optimization (PSO), grey wolf optimizer (GWO), and whale optimization (WOA) were explored and compared to the DTACO algorithm. The basic multilayer perceptron (MLP) regressor model, the support vector regression (SVR) model, and the random forest (RF) regressor model were contrasted with the optimal ensemble DTACO-based model that was proposed. In order to assess the consistency of the DTACO-based model that was developed, the statistical research made use of Wilcoxon’s rank-sum and ANOVA tests.

## 1. Introduction

Metamaterials have been addressed in a lot of research in various fields. Applications of metamaterials also include metamaterial antenna, in which metamaterials are utilized to improve their performance. The size of the electromagnetic antenna affects its radiation loss and quality factor. However, a tiny antenna with low cost and high efficiency is preferred for an integrated antenna. The metamaterial can create small antennas with improved bandwidth and gain. It can also help to minimize their electrical size and increase their directivity. Metamaterial antennas can solve the bandwidth limitation of small antennas. Simulation software is employed to estimate the effect of metamaterial on the antenna characteristics, including its gain and bandwidth. During simulation, the metamaterial antenna is adjusted by trial and error to fulfill the expected parameters. However, this process can take much longer than expected. Machine learning (ML) and algorithms can be used to forecast antenna characteristics as an alternative to simulation software. ML is a branch of artificial intelligence extensively used in different engineering applications in making decisions or predictions. This study addresses the challenge of using optimized ML models to forecast the metamaterial antenna’s gain and bandwidth [1,2].

Metamaterial antenna has been studied extensively in the literature, as it has unusual properties [3,4]. These properties enhance the abilities of the original material and their engagement in the industry [5]. Metamaterial antennas are derived from a field of science engineering known as computational electromagnetics. Computational electromagnetics is based on optimizing methods and computation for designing antennas. However, traditional design paradigms comprising model designs, parameter sweep, trial-and-error methods, and optimization algorithms are time-consuming and use a large amount of computing resources. Furthermore, if the design requirements change, simulations must be rerun, preventing the scientists from focusing on their actual demands. As a result, we have considered machine learning to fill in the gaps in our search for a quick, efficient, and automated design strategy.

Over the past few years, there has been an increase in research focused on combining machine learning techniques with metaheuristics to solve combinatorial optimization problems. This integration aims to enhance the efficiency, effectiveness, and resilience of metaheuristics during their search process and ultimately improve their performance in terms of solution quality, convergence rate, and robustness. In addition, there are several techniques developed to tackle the different optimization problems [6,7,8,9]. Optimization problems can be found in almost any field of study [10]. Some of the most popular areas are medicine [11], engineering problems [12,13,14], image processing [15], feature selection [16], etc. [17,18].

Recently, the utilization of ML in computational electromagnetics has attracted the research community’s attention [19,20,21,22]. The most important benefit of ML-aided electromagnetics lies in the ability to create an underlying relationship between the system input parameters and the desired outcomes; consequently, the computational burden in experimental real-time processing is shifted to the offline training stage [23]. The application of ML in metamaterial antenna design is a promising approach to deal with its high complexity and computational burden [24,25]. In [26], a joint design for antenna selection was proposed, using two deep learning models to forecast the selected antennas and estimate the hybrid beamformers. Another work utilized KNN and SVM for multiclass classification of the antenna selection in multiple-input, multiple-output (MIMO) systems [27].

In [25], ANN was employed to predict the selected antennas having a minimum signal-to-noise ratio (SNR) among the users. The authors of [24] used SVM and naive Bayes as a hybrid ML model for forecasting a secure-based antenna on the wiretap channel. In [21], SVM was utilized with several antennas in multiuser communication systems. The authors suggested an antenna allocation system based on the support vector machine. In [20], the authors built a support vector regression model trained on data collected from a microwave simulator to design the feed in a rectangular patch antenna. In this research, the performance of the ensemble ML approach is investigated in forecasting the bandwidth of the metamaterial antenna. An optimization technique is utilized to estimate the optimal weights of the learning model. Furthermore, a binary version of the proposed algorithm is introduced to select the best features from the input dataset.

There are several ML algorithms, such as k-nearest neighbor (KNN) [28], artificial neural network (ANN) [27], decision tree [29], and support vector machine (SVM). The main concept of these algorithms is building a learning model that can generalize and predict unseen data. For example, ANN is an intelligent learning model that simulates the biological nervous system [27]. One of the most common architectures of feedforward ANN is called multilayer perceptron (MLP), which comprises an input layer, a set of hidden layers, and an output layer. Ensemble ML is based on combining two or more ML models to improve the performance of the base ML models [30], including ANN, KNN, and SVM. The main principle of ensemble learning is estimating an output by averaging the output values of the base ML models. In average-based ensemble learning, every base model contributes the same weight to the computation. This may result in an undesired performance of the ensemble methods. An efficient performance can be yielded by using an optimization technique to calculate the weights of the base models.

**Figure 1:**
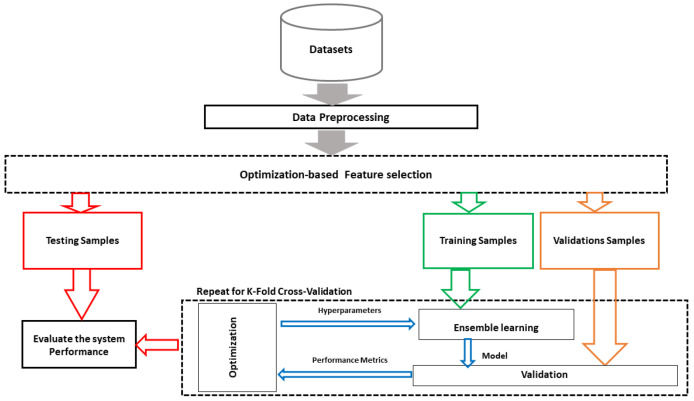
The proposed framework for forecasting gain and bandwidth of metamaterial antenna.

The proposed system is illustrated in Figure 1. The system includes three main stages: data preprocessing for inputting the missing values and normalization of the data values; feature selection; and the regression stage through optimized ensemble learning for forecasting the bandwidth of the metamaterial antenna. The proposed framework is applied to publicly available metamaterial forecasting datasets from the Kaggle platform [31].

In this study, machine learning is incorporated into antenna design. The electromagnetic properties of an antenna that have been established via a series of experimental simulations are used to train a machine learning system. The ML algorithm helps design a metamaterial antenna that delivers the closest results based on the designer’s requirements. The following objectives will be yielded through this study:

- Developing an ML model using the ensemble learning approach for forecasting the bandwidth of the metamaterial antenna.
- Developing metaheuristic optimization techniques to establish an efficient ensemble ML model.
- Developing an optimization algorithm to select the significant features from the input dataset.
- Comparing the performance of the proposed model with the state-of-the-art ML models in forecasting bandwidth of the metamaterial antenna.

The following structure can be used for the remaining parts of the paper. Section 2 presents the related work. In Section 3, we will go over a general summary of the materials and procedures. Section 4 explains the mathematical methodology for estimating the bandwidth of metamaterial antennas using the DTACO model in depth. In Section 5, we will discuss some experimental simulations and various situations for comparison. Section 6 summarizes the advantages and disadvantages of the suggested method when it is used in real life. Section 7 of the paper is where the conclusion and future work can be found.

## 2. Related Work

The frequency selective surface (FSS)-based filtering antenna Filtenna was given computationally efficient optimization recommendations in [32]. The Filtenna enhances and filters signals at a certain frequency. It is challenging to build Filtenna FSS elements because of the numerous variables and intricate interrelations that affect scattering responses. An accurate model of unit cell behavior is created by the authors using a deep learning method called modified multilayer perceptron (M2LP). Filtenna FSS elements are optimized by the M2LP model. The proposed approach reduces the computational cost of optimization by 90% when compared to direct electromagnetic (EM)-driven design. An experimental Filtenna prototype demonstrates the efficacy of the method. Without incurring additional computing overhead, the unit cell model may generate FSS and Filtenna in many frequency ranges. The authors of [33] suggested limiting antenna response sensitivity updates to dominant directions in parameter space to hasten antenna tuning. The dominant directions are determined by problem-specific information, or more precisely, how estimated antenna characteristics vary when moving through the one-dimensional affine subspaces encompassed by these directions. The processing costs of full-wave electromagnetic (EM) simulations used in antenna optimization are decreased via local optimization. The results show a 60% speedup over reference approaches without sacrificing quality. The method is evaluated against accelerated versions of trust region algorithms and various antenna topologies.

It is essential to optimize the features of the antenna system. While parametric analyses are common, more exact numerical techniques are required for optimal designs in complex problems with multiple variables, goals, and constraints. The reliability and cost of computation for EM-driven optimization are problematic. Without a solid starting point or multimodal objective function, local numerical algorithms may find it challenging to identify effective designs for EM simulations, which are expensive to run. The reliability of an antenna can be increased by following a recent strategy that suggests matching design objectives (such as center frequencies) with the antenna’s actual operational parameters at each design iteration [34]. With this modification, a local search is now feasible, and the objectives are gradually being brought closer to the initial targets. Through the use of a specification management system and variable-resolution optimization framework, this research proposes a trustworthy and economical antenna-tuning technique. Depending on the discrepancy between the actual and desired operating conditions and algorithm convergence, the algorithm adaptively modifies EM model fidelity. When compared to a single-fidelity method, starting the search with the lowest-fidelity model and gradually raising it results in computational cost savings of roughly 60%.

The work in [35] addressed reflectarray (RA) design difficulties, which have advantages over traditional antenna arrays but narrow bandwidths and losses. Inverse surrogate modeling reduces computing costs for the independent adjustment of many unit cells in an alternate RA design technique. A few reference reflection phase-optimized anchor points alter the unit cells. Anchor point optimization uses minimum-volume unit cell regularization for solution uniqueness. The provided method lowers RA design computation to a few dozen cell EM analyses. The method is illustrated and tested. A fully adaptive regression model (FARM) was proposed in [36] for accurate transistor scattering and noise parameter modeling utilizing artificial neural networks (ANNs), particularly deep learning. Characteristics, designable parameters, biasing conditions, and frequency are complex, making transistor modeling difficult. A tree Parzen estimator automatically determines all network components and processing functions in the FARM technique to match input data and network architecture. Three microwave transistors are used to validate the strategy, which outperforms ANN-based methods in modeling accuracy.

Microwave component design increasingly relies on numerical optimization. Circuit theory techniques can produce good beginning designs, but electromagnetic cross-coupling and radiation losses require fine parameter tweaking. Gradient-based EM-driven design closure processes work well when the initial design is near the optimum. If the starting design is not optimal, the search process may converge to a poor local optimum. Simulation-based optimization is computationally expensive. Research in [37] proposed a new parameter-tuning method using variable-resolution EM models and a recently published design specification management methodology. The design specification management approach automates design objective modification during the search process, boosting robustness to bad starting points. Algorithm convergence and performance specification disagreement determine simulation model fidelity. Lower-resolution EM simulations in the early optimization phase can save up to 60% computationally compared to a gradient-based search with design specification management and numerical derivatives. Three microstrip circuit tests demonstrate computational speedup without compromising design quality.

Surrogate modeling is preferred for difficult antenna design projects that need expensive full-wave electromagnetic simulations. Traditional metamodeling methodologies cannot handle nonlinear antenna characteristics across a large range of system parameters due to the curse of dimensionality. Performance-driven modeling frameworks that build surrogates from antenna performance numbers rather than geometric factors can overcome this issue [38]. This method dramatically reduces model setup costs without losing design utility. This study provides a domain confinement-based variable-fidelity electromagnetic simulation modeling framework. The final surrogate is generated using co-kriging, which combines simulation data of diverse fidelities. Three microstrip antennas validate this approach, showing reliable models with much lower CPU costs than conventional and performance-driven modeling methods.

Quantifying fabrication tolerances and uncertainties in antenna design helps antennas resist manufacturing errors and material parameter fluctuations. Industrial settings require this. Geometric parameters can degrade electrical and field properties, causing frequency shifts and impedance matching. Maximizing manufacturing yield requires computationally intensive full-wave electromagnetic analysis to improve antenna performance in the presence of uncertainty. The curse of dimensionality has plagued surrogate modeling methods used to overcome these issues [39]. This work provides a low-cost antenna yield optimization method. It carefully defines the domain of the statistical analysis metamodel, which consists of a few influential directions controlling antenna responses in the relevant frequency bands. Circuit response variability assessment automates these directions. A small domain volume reduces surrogate model setup cost while improving yield. Three antenna topologies validate the proposed strategy, which outperforms multiple benchmark methods with surrogate models. Electromagnetic-driven Monte Carlo simulations prove the yield optimization’s reliability.

Adaptive algorithms dynamically adjust their search strategies based on problem characteristics or optimization progress. Hybrid algorithms combine multiple optimization techniques to leverage their strengths and compensate for their weaknesses. Considering adaptive or hybrid algorithms can enhance the optimization process by adapting to the specific requirements and challenges of the metamaterial design problem. Metaheuristic algorithms such as dipper-throated optimization and ant colony optimization can effectively handle complex optimization problems such as metamaterial design. These algorithms provide a broader exploration of the design space and can help find global optima.

## 3. Materials and Methods

### 3.1. Dipper-Throated Optimization (DTO)

Dipper-throated passerines are rare. They dive, hunt, and swim well. Their flexible, tiny wings let them fly straight and rapidly without glides or pauses. The dipper-throated bird (DTO) method assumes birds fly and swim to find food, with $N_{fs}$ denoting the number of birds. Bird locations are $N_{fs}$ and velocities are BV. BP and BV are represented as follows [40]:

(1) $$BP=\left[\begin{array}{ccccc} BP_{1,1} & BP_{1,2} & BP_{1,3} & \cdots & BP_{1,d}\\ BP_{2,1} & BP_{2,2} & BP_{2,3} & \cdots & BP_{2,d}\\ BP_{3,1} & BP_{3,2} & BP_{3,3} & \cdots & BP_{3,d}\\ \cdots & \cdots & \cdots & \cdots & \cdots \\ BP_{n,1} & BP_{n,2} & BP_{n,3} & \cdots & BP_{n,d} \end{array}\right]$$

where $BP_{i,j}$ is the *i*th bird position in *j*th dimension. The agents are considered initially uniformly distributed.

(2) $$BV=\left[\begin{array}{ccccc} BV_{1,1} & BV_{1,2} & BV_{1,3} & \cdots & BV_{1,d}\\ BV_{2,1} & BV_{2,2} & BV_{2,3} & \cdots & BV_{2,d}\\ BV_{3,1} & BV_{3,2} & BV_{3,3} & \cdots & BV_{3,d}\\ \cdots & \cdots & \cdots & \cdots & \cdots \\ BV_{n,1} & BV_{n,2} & BV_{n,3} & \cdots & BV_{n,d} \end{array}\right]$$

where $BV_{i,j}$ is the *i*th velocity of bird in *j*th dimension. The values of the objective function, $f_{n}$, are determined as follows:

(3) $$f=\left[\begin{array}{c} f_{1}\left(BP_{1,1},BP_{1,2},BP_{1,3},\cdots ,BP_{1,d}\right)\\ f_{2}\left(BP_{2,1},BP_{2,2},BP_{2,3},\cdots ,BP_{2,d}\right)\\ f_{3}\left(BP_{3,1},BP_{3,2},BP_{3,3},\cdots ,BP_{3,d}\right)\\ \cdots \\ f_{n}\left(BP_{n,1},BP_{n,2},BP_{n,3},\cdots ,BP_{n,d}\right) \end{array}\right]$$

Then, the objective function values are ordered from lowest to highest in increasing order. It has been determined that $BP_{best}$ is the first best solution. It is anticipated that the remaining responses will pertain to typical birds. $BP_{nd}$ is an abbreviation for follower birds. It has been decided that the $BP_{Gbest}$ solution is the best one possible overall.

The location of the swimming bird shifts as it swims, as follows:

(4) $$BP_{nd}\left(t+1\right)=BP_{best}\left(t\right)-C_{1}.\left|C_{2}.BP_{best}\left(t\right)-BP_{nd}\left(t\right)\right|$$

where the $C_{1}$ and $C_{2}$ parameters are determined as $C_{1}=2c.r_{1}-c$ and $C_{2}=2r_{1}$ for $c=2(1-{\left(\frac{t}{T_{max}}\right)}^{2})$, which is updated from 2 to 0 exponentially. $r_{1}$ is updated randomly within [0,1] and $T_{max}$ is maximum iterations.

The positions of the flying birds are updated as follows:

(5) $$BP_{nd}\left(t+1\right)=BP_{nd}\left(t\right)+BV\left(t+1\right)$$

The velocities of the flying birds are changed as follows:

(6) $$\begin{array}{cc} BV\left(t+1\right)= & C_{3}BV\left(t\right)+C_{4}r_{2}\left(BP_{best}\left(t\right)-BP_{nd}\left(t\right)\right)+\\ & C_{5}r_{2}\left(BP_{Gbest}-BP_{nd}\left(t\right)\right) \end{array}$$

where $C_{3}$ is a weight value, $C_{4}$ and $C_{5}$ are constants. $r_{2}$ is updated randomly within [0,1]. The DTO algorithm is broken down into its component parts and detailed in Algorithm 1.
**Algorithm 1 **DTO Algorithm  1:**Initialize** birds’ positions as $BP_{i}\left(i=1,2,...,n\right)$, birds’ velocities as $BV_{i}\left(i=1,2,...,n\right)$, iterations $T_{max}$, objective function $f_{n}$, other DTO parameters, t=1  2:**Calculate** $f_{n}$ for each bird $BP_{i}$  3:**Find** the best bird $BP_{best}$  4:**while **$t\leq T_{max}$ **do**  5:   **for** (i=1:i<n+1) **do**  6:       **if** (R<0.5) **then**  7:          **Update** swimming birds’ positions as               $BP_{nd}\left(t+1\right)=BP_{best}\left(t\right)-C_{1}.\left|C_{2}.BP_{best}\left(t\right)-BP_{nd}\left(t\right)\right|$  8:       **else**  9:          **Update** flying birds’ velocities as               $BV\left(t+1\right)=C_{3}BV\left(t\right)+C_{4}r_{2}\left(BP_{best}\left(t\right)-BP_{nd}\left(t\right)\right)+C_{5}r_{2}\left(BP_{Gbest}-BP_{nd}\left(t\right)\right)$10:          **Update** flying birds’ positions as               $BP_{nd}\left(t+1\right)=BP_{nd}\left(t\right)+BV\left(t+1\right)$11:       **end if**12:   **end for**13:   **Update** $f_{n}$ for each bird $BP_{i}$14:   **Update** parameters, t=t+115:   **Update** the best bird $BP_{best}$16:   **Set** $BP_{Gbest}$ = $BP_{best}$17:**end while**18:**Return **$BP_{Gbest}$

### 3.2. Ant Colony Optimization (ACO)

Ant foraging inspired the ACO algorithm. Ant colonies can always determine the best route from the nest to the food supply. While foraging, ants generate pheromones that other ants can detect. The shorter path has more pheromones since they evaporate over time. Thus, the ant swarm can choose an optimal path and migrate toward high pheromone intensity [41].

ACO parameters, including maximum iterations $T_{max}$, number of ants *m*, pheromone evaporation factor ρ, heuristic factor α, predicted heuristic factor β, and intensity value *Q*, are initialized. Path routing memory matrices, heuristic information, and pheromones should also be initialized. ACO involves choosing the best path. ACO uses roulette wheel selection. This strategy bases selection on fitness. In classic ACO, the probability selection rule for the *k*th ant traveling from *i*th to *j*th position is defined as follows:

(7) $$\begin{array}{cc} \; & P_{ij}^{m}=\left\{\begin{array}{cc} \frac{{\left[\tau \left(i,j\right)\right]}^{\alpha }*{\left[\eta \left(i,j\right)\right]}^{\beta }}{\sum_{S\in J_{m}\left(i\right)}{\left[\tau \left(i,S\right)\right]}^{\alpha }*{\left[\eta \left(i,S\right)\right]}^{\beta }}, & \left(i,j\right)\in J_{m}\\ 0 & otherwise \end{array}\right. \end{array}$$

where for ant *m*, $J_{m}$ is the selectable grid collection in the next iteration. The $P_{ij}^{m}$ parameter indicates the transition probability for every optional path from *i*th to *j*th position. τ(i,j) indicates the pheromone concentration value on point (i,j) and η(i,j) is the heuristic information visibility. η(i,j) is calculated as $\frac{1}{d_{ij}}$, which is the Euclidean distance from *i*th to *j*th point.

After each ant has constructed a path from starting point to the destination point, the concentration of pheromones on each edge of the path will be changed using the overall distance traveled by the path. After that, the global pheromone concentration will be brought up to date following the completion of an iterative search by all of the ants. The updating rule for the concentration of pheromones is displayed as follows.

(8) $$\tau_{t+1}^{m}\left(i,j\right)=\left(1-\rho \right)*\tau_{t}^{m}\left(i,j\right)+\sum_{m=1}^{M}\Delta \tau_{t}^{m}\left(i,j\right),$$

(9) $$\begin{array}{cc} \; & \Delta \tau_{t}^{m}\left(i,j\right)=\left\{\begin{array}{cc} \frac{Q}{L_{m}}, & if\;ant\;m\;travels\;from\;node\;i\;to\;node\;j\\ 0 & otherwise \end{array}\right. \end{array}$$

where $\tau_{t}^{m}\left(i,j\right)$ indicates the pheromone concentration from the *i*th to *j*th point, while $\Delta \tau_{t}^{m}\left(i,j\right)$ is the pheromone concentration variation. ρ is the global pheromone evaporation factor with a value in [0, 1]. (1−ρ) represents the pheromone residual coefficient. *Q* indicates pheromone intensity, which is a constant, and $L_{m}$ is the *m*th ant total length in the current iteration. The ACO algorithm is broken down into steps and detailed in Algorithm 2.
**Algorithm 2 **ACO Algorithm  1:**Initialize** ants’ positions, concentration of pheromones as $\tau_{i,j}$, with *m* ants, iterations $T_{max}$, objective function $f_{n}$, other ACO parameters, t=1  2:**Calculate**$f_{n}$ for each ant  3:**while** $t\leq T_{max}$ **do**  4:   **for** (i=1:i<m+1) **do**  5:         **Update** the probability as         $P_{ij}^{m}=\frac{{\left[\tau \left(i,j\right)\right]}^{\alpha }*{\left[\eta \left(i,j\right)\right]}^{\beta }}{\sum_{S\in J_{m}\left(i\right)}{\left[\tau \left(i,S\right)\right]}^{\alpha }*{\left[\eta \left(i,S\right)\right]}^{\beta }}$  6:         **Update** ants’ positions; each ant moves from point *i*th to point *j*th based on the probability of its movements.  7:         **Update** the pheromone concentration variation as         $\Delta \tau_{t}^{m}\left(i,j\right)$  8:         **Update** the pheromone concentration as         $\tau_{t+1}^{m}\left(i,j\right)=\left(1-\rho \right)*\tau_{t}^{m}\left(i,j\right)+\sum_{m=1}^{M}\Delta \tau_{t}^{m}\left(i,j\right)$  9:   **end for**10:   **Update** $f_{n}$ for each ant11:   **Update** ACO parameters, t=t+112:   **Update** the best ant13:**end while**14:**Return** best ant position

## 4. Proposed Methodology

### 4.1. Proposed DTACO Algorithm

Algorithm 3 presents the suggested dipper-throated-based ant colony optimization (DTACO) algorithm step by step. The DTACO algorithm balances the benefits of the DTO and ACO algorithms while addressing their drawbacks to produce the best overall result. The beginning steps of the algorithm involve setting the positions of certain specified *n* agents $x_{i}\left(i=1,2,\cdots ,n\right)$ and their velocities $v_{i}\left(i=1,2,\cdots ,n\right)$. Additionally, the maximum number of permissible iterations for the execution process is set by this $T_{max}$, objective function $f_{n}$, and the DTO and ACO parameters. The term $R_{DTACO}$ indicates a random value between 0 and 1.

If $R_{DTACO}>0.5$, the DTACO algorithm updates the agents’ positions and agents’ velocities as follows. The positions of the swimming agent will be updated if R<0.5 by

(10) $$x\left(t+1\right)=x_{best}\left(t\right)-C_{1}.\left|C_{2}.x_{best}\left(t\right)-x\left(t\right)\right|$$

If R≥0.5, the agents are considered flying agents, and then the positions will be changed as

(11) x(t+1)=x(t)+v(t+1)

where v(t+1), and updated velocity is calculated for each agent as follows:

(12) $$v\left(t+1\right)=C_{3}v\left(t\right)+C_{4}r_{2}\left(x_{best}\left(t\right)-x_{nd}\left(t\right)\right)+C_{5}r_{2}\left(x_{Gbest}-x\left(t\right)\right)$$

If $R_{DTACO}\leq 0.5$, the DTACO algorithm will update the probability selection rule for the *k*th ant traveling from *i*th to *j*th position as follows.

(13) $$P_{ij}^{m}=\frac{{\left[\tau \left(i,j\right)\right]}^{\alpha }*{\left[\eta \left(i,j\right)\right]}^{\beta }}{\sum_{S\in J_{m}\left(i\right)}{\left[\tau \left(i,S\right)\right]}^{\alpha }*{\left[\eta \left(i,S\right)\right]}^{\beta }}$$

where $P_{ij}^{m}$ is the transition probability for every optional path from the *i*th to the *j*th position. τ(i,j) is the pheromone concentration value on point (i,j) and η(i,j) is the heuristic information visibility.
**Algorithm 3 **Proposed DTACO Algorithm  1:**Initialize** agents’ positions, $x_{i}\left(i=1,2,\cdots ,m\right)$, with *m* agents, agents’ velocities, $v_{i}\left(i=1,2,\cdots ,m\right)$, iterations $T_{max}$, objective function $f_{n}$, parameters, $R_{DTACO}$, t=1  2:**Obtain **$f_{n}$ for agents  3:**Find** the best agent $x_{best}$  4:**while **t≤Tmax** do**  5:   **if** ($R_{DTACO}>0.5$) **then**  6:     **for** (i=1:i<m+1) **do**  7:        **if** (R<0.5) **then**  8:            **Update** the swimming agent position by            $x\left(t+1\right)=x_{best}\left(t\right)-C_{1}.\left|C_{2}.x_{best}\left(t\right)-x\left(t\right)\right|$  9:        **else**10:            **Update** the flying agent velocity by            $v\left(t+1\right)=C_{3}v\left(t\right)+C_{4}r_{2}\left(x_{best}\left(t\right)-x\left(t\right)\right)+C_{5}r_{2}\left(x_{Gbest}-x\left(t\right)\right)$11:            **Update** the flying agent position by            x(t+1)=x(t)+v(t+1)12:        **end if**13:     **end for**14:   **else**15:     **for** (i=1:i<m+1) **do**16:        **Update** the probability as        $P_{ij}^{m}=\frac{{\left[\tau \left(i,j\right)\right]}^{\alpha }*{\left[\eta \left(i,j\right)\right]}^{\beta }}{\sum_{S\in J_{m}\left(i\right)}{\left[\tau \left(i,S\right)\right]}^{\alpha }*{\left[\eta \left(i,S\right)\right]}^{\beta }}$17:        **Update** ants’ positions; each ant moves from point *i*th to point *j*th based on the probability of its movements.18:        **Update** the pheromone concentration variation as        $\Delta \tau_{t}^{m}\left(i,j\right)$19:        **Update** the pheromone concentration as        $\tau_{t+1}^{m}\left(i,j\right)=\left(1-\rho \right)*\tau_{t}^{m}\left(i,j\right)+\sum_{m=1}^{M}\Delta \tau_{t}^{m}\left(i,j\right)$20:     **end for**21:   **end if**22:   **Obtain** $f_{n}$ for agents23:   **Update** parameters, t=t+124:   **Find** best agent $x_{best}$25:   **Set** $x_{Gbest}$ = $x_{best}$26:**end while**27:**Return** best agent $x_{Gbest}$

After constructing a path from the starting point to the destination point by each ant, the concentration of pheromones on each edge of the path is changed using the overall distance traveled by the path. After completing an iterative search by all ants, the global pheromone concentration will be updated as follows:

(14) $$\tau_{t+1}^{m}\left(i,j\right)=\left(1-\rho \right)*\tau_{t}^{m}\left(i,j\right)+\sum_{m=1}^{M}\Delta \tau_{t}^{m}\left(i,j\right),$$

where $\Delta \tau_{t}^{m}\left(i,j\right)$ is the pheromone concentration variation. ρ is the global pheromone evaporation factor with a value in [0, 1]. (1−ρ) represents the pheromone residual coefficient. *Q* indicates pheromone intensity, which is a constant, and $L_{m}$ is the *m*th ant total length in the current iteration.

The following is an expression of the computational difficulty posed by the DTACO algorithm within the context of this work. The level of complexity is defined as follows for iterations with a maximum of $t_{max}$ and *m* agents:

- Initialize parameters of the DTACO algorithm: *O*(1).
- Calculate $f_{n}$ for each agent: *O*(*m*).
- Find the best agent: *O* (*m*).
- Update agents’ positions: *O*($t_{max}\times m$).
- Update agents’ velocities: *O*($t_{max}\times m$).
- Update agents’ positions: *O*($t_{max}\times m$).
- Update probability: *O*($t_{max}\times m$).
- Update agents’ positions: *O*($t_{max}\times m$).
- Update pheromone concentration variation: *O*($t_{max}\times m$).
- Update pheromone concentration: *O*($t_{max}\times m$).
- Update parameters, t=t+1: *O*($t_{max}$).
- Obtain best agent $x_{best}$: *O*($t_{max}$).
- Set $x_{Gbest}$ = $x_{best}$: *O*($t_{max}$).
- Obtain global best agent $x_{Gbest}$: *O*(1)

As a result of the above examination of the DTACO method, the complexity of the calculation has been determined to be *O*($t_{max}\times m$), but it will be *O*($t_{max}\times m\times d$) for the *d* dimension.

### 4.2. Proposed Binary DTACO Algorithm

In the event that there are problems with feature selection, the solutions produced by the DTACO algorithm will be purely binary, taking the form of values of 0 or 1. In order to make the process of selecting features from the dataset more manageable, the continuous values returned by the proposed DTACO method will be converted to a binary representation of [0, 1]. This investigation makes use of an equation that is derived based on the Sigmoid function and is shown below [40]:

(15) $$\begin{array}{cc} \; & x\left(t+1\right)=\left\{\begin{array}{cc} 1 & \mathrm{if}\;Sigmoid(n)\geq 0.5\\ 0 & otherwise \end{array}\right.,Sigmoid\left(n\right)=\frac{1}{1+e^{-10(n-0.5)}}, \end{array}$$

where x(t+1) represents a binary solution. The Sigmoid function scales the solutions to binary ones. For Sigmoid(n)≥0.5, the value will be 1; otherwise, the value will be 0. The *n* indicates the proposed algorithm’s solution. The algorithm known as binary DTACO (bDTACO) is outlined in further detail in Algorithm 4.
**Algorithm 4 **Proposed Binary DTACO Algorithm  1:**Initialize** parameters  2:**Obtain **$f_{n}$ for agents  3:**Find** best agent  4:**Change** solutions to binary [0,1]  5:**while **t≤Tmax** do**  6:   **if** ($R_{DTACO}>0.5$) **then**  7:     **for** (i=1:i<m+1) **do**  8:        **if** (R<0.5) **then**  9:          **Update** the swimming agent position10:        **else**11:          **Update** the flying agent velocity12:          **Update** the flying agent position13:        **end if**14:     **end for**15:   **else**16:     **for** (i=1:i<m+1) **do**17:        **Update** the probability18:        **Update** ants’ positions19:        **Update** the pheromone concentration variation20:        **Update** the pheromone concentration21:     **end for**22:   **end if**23:   **Obtain** $f_{n}$ for agents24:   **Update** parameters25:   **Find** best agent $x_{best}$26:   **Set** $x_{Gbest}$ = $x_{best}$27:   **Change** updated solution to binary by Equation (15)28:**end while**29:**Return** best agent $x_{Gbest}$

## 5. Experimental Results

The entire purpose of this part is to provide a thorough examination of the investigation’s findings. The investigations were carried out in two different contexts. The proposed binary DTACO algorithm’s feature selection capabilities for the dataset under test are covered in the first scenario, and the algorithm’s regression capabilities are demonstrated in the second scenario. The DTACO algorithm was analyzed and compared to other algorithms that are considered to be state-of-the-art, including DTO [40], ACO [41], particle swarm optimization (PSO) [42], the grey wolf optimizer (GWO) [43], the genetic algorithm (GA) [43], and the whale optimization algorithm (WOA) [44]. Both scenarios are described below. A presentation of the DTACO algorithm configuration can be found in Table 1. This presentation includes all of the experiment’s relevant parameters. It is essential to provide details about the numerous parameters that will be used to determine the behavior and performance of the algorithm. These settings include population size (number of agents), the termination criterion (number of iterations), and other important characteristics for optimization m to select the significant features from the input dataset.

Table 2 presents the comparative algorithms’ setup. To evaluate optimization techniques and parameters fairly, many aspects were considered. First, we considered the search space size, constraints, and objective function. Choosing a problem-specific algorithm can improve performance. Second, parameter choice affects algorithm performance. We considered convergence speed and exploration–exploitation trade-offs while tuning parameters for the issue and method. For fair comparisons, ten runs with varied random seeds were applied, statistical analysis was performed, and an appropriate dataset was tested. A fair comparison of DTO, ACO, GWO, PSO, GA, and WOA optimization algorithms and parameter selection yielded meaningful insights and informed decision making. The computational budget was established based on the number of function calls made during optimization. Each optimizer was run ten times for 80 iterations, and the number of search agents was set to 10. Setting a specific computational budget ensured that all the compared algorithms had an equal opportunity to explore and exploit the search space within the given limitations. This approach allows for a fair and standardized evaluation, facilitating meaningful comparisons between optimization algorithms.

### 5.1. Dataset

**Figure 2:**
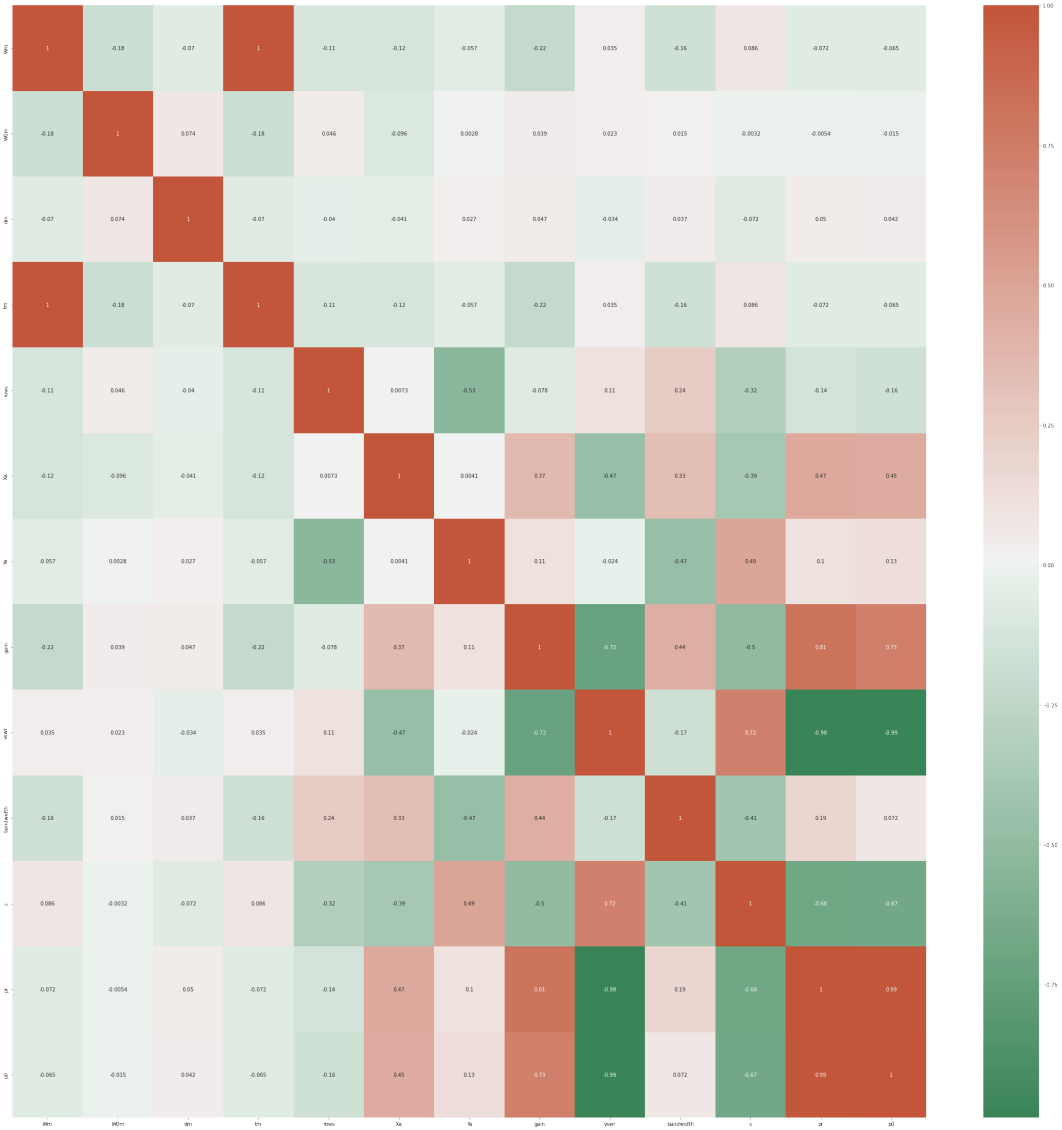
Heat map of the metamaterial antenna forecasting dataset.

The dataset is freely available and can be utilized in constructing a machine learning model for improved radiation efficiency of an antenna [31]. The dimensions of a patch antenna, the dimensions of the slots in the patch antenna, the operating frequency, and finally, the matching S11 parameter are all included in this dataset. The HFSS program was utilized in the construction of the antenna as well as the collection of the dataset. Ansys HFSS is a software tool utilized to design and simulate high-frequency electronic devices. This 3D electromagnetic (EM) simulation software is specifically tailored for creating and evaluating various products, including antennas, antenna arrays, filters, connectors, and printed circuit boards. Its primary purpose is to provide accurate modeling and analysis capabilities for the development of these high-frequency electronic systems. The radiation frequency of the tested dataset is maintained at 2.4 GHz, making it compatible with Bluetooth and wireless local area network (WLAN) operations. Figure 2 presents a heat map that can be used to gain insight into the manner in which the variables are connected.

### 5.2. Feature Selection Scenario

When selecting features from the dataset that was put through its paces, the binary implementation of the DTACO method that was proposed is the one that comes into play. In the first scenario, a discussion of the outcomes of the feature selection carried out using the DTACO algorithm described in this paper is included. The binary DTACO (bDTACO) method is analyzed and contrasted with the binary DTO (bDTO), binary ACO (bACO), binary PSO (bPSO), binary GWO (bGWO), and binary GA (bGA).

With the assistance of the objective equation, also known as $f_{n}$, the binary DTACO method is able to determine the level of quality possessed by a given solution. In the equation that follows, fn is used as a variable in the expressions for a number of selected features (*v*), the total number of features (*V*), and a classifier’s error rate (Err).

(16) $$f_{n}=h_{1}Err+h_{2}\frac{\left|v\right|}{\left|V\right|}$$

where the significance of the provided feature to the population is indicated by the formula $h_{2}=1-h_{1}$, and the value of $h_{1}$ might fall anywhere in the range [0, 1]. If it is possible to supply a subset of features that are capable of providing a low classification error rate, then the approach can be called acceptable. The k-nearest neighbor technique, also referred to as kNN, is a straightforward classification method that is frequently put into practice. In this method, the employment of the k-nearest neighbor classifier assures that the chosen attributes are of good quality. The only criterion that is used in the process of determining classifiers is the distance that is considered to be the shortest between the query instance and the training instances. This experiment does not make use of any models for the K-nearest neighbor technique in any way.

The effectiveness of the suggested strategy for feature selection is evaluated in accordance with the standards presented in Table 3. This table also includes a column labeled "M" that contains the total number of iterations performed by both the proposed optimizer and its rivals. The symbol $S_{j}^{*}$ is used to designate the best solution, and the size of the best solution vector is denoted by the value $size(S_{j}^{*})$. The total number of points for the test set is denoted by the letter *N*. The predicted values is denoted by the term $\hat{V_{n}}$, while the actual values are denoted by the term $V_{n}$.

The results of feature selection using the proposed and compared algorithms are presented in Table 4. As shown in Table 1, these outcomes are based on 80 iterations over 10 runs for 10 agents. With an average error of (0.5027) and a standard deviation of (0.4055), the given bDTACO technique performed as expected. The next best algorithms are bDTO, with a score of (0.5265); bACO, with a score of (0.5308); bGWO, with a score of (0.5472); bGA, with a score of (0.5694); bWOA, with a score of (0.5708); and finally, bPSO, with a score of (0.571), which accomplish the lowest minimal average error in the feature selection process for the data that have been evaluated. When it comes to feature selection, the bPSO algorithm is the weakest one available.

**Figure 3:**
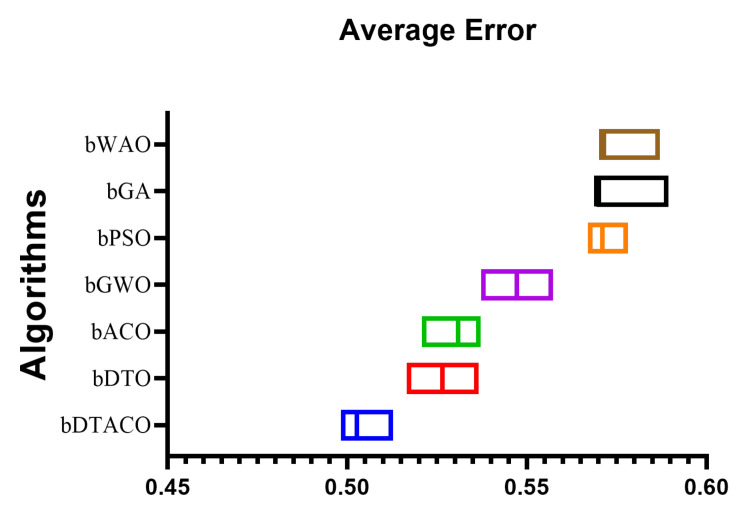
The proposed bDTACO method, together with the bDTO, bACO, bPSOm bGWO, bGA, and bWOA algorithms, are compared using a box plot that is based on the average error for each algorithm.

**Figure 4:**
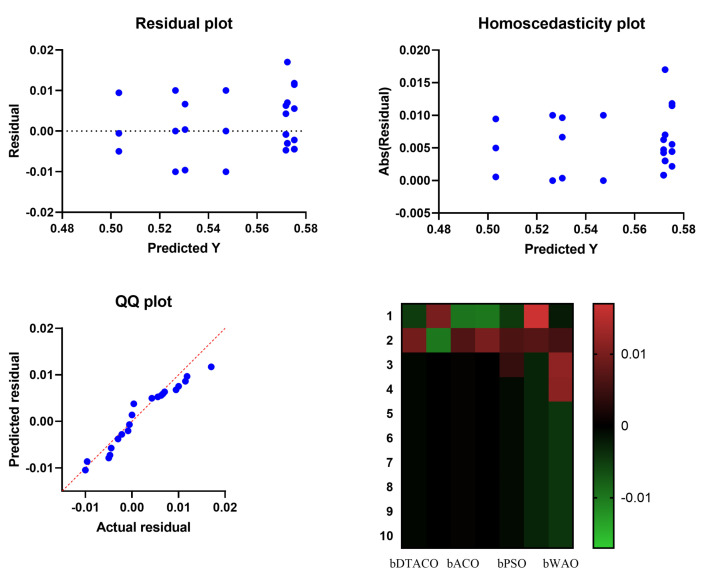
Quantile–quantile plots and residual plots, as well as a heat map, for the bDTACO that was presented and the methods that were compared.

Figure 3 displays the box plot that was generated based on the average error for the bDTACO algorithm, as well as the bDTO algorithm, the bACO algorithm, the bPSO algorithm, the bGWO algorithm, the bGA algorithm, and the bWOA algorithm. The quality of the bDTACO algorithm, as determined by utilizing the objective function described in Equation (16), is displayed in the figure. Figure 4 presents the quantile–quantile (QQ) plots, residual plots, and heat map for both the given bDTACO and the methods that were compared for the data that were analyzed. These plots show the relationship between the data and the quantiles and quantile differences.

This statistical analysis uses one-way ANOVA and Wilcoxon signed-rank tests to determine the average error of the suggested binary DTACO algorithm. The Wilcoxon test determines *p*-values for comparing the suggested approach to other methods. This statistical test can assess if the suggested algorithm outperforms other algorithms with a *p*-value of less than 0.05. The analysis of variance (ANOVA) test was also performed to determine if the suggested algorithm differed significantly from the others. Table 5 shows the ANOVA test results for the proposed algorithm vs. the methods compared, and Table 6 shows the Wilcoxon signed-rank test results. The statistical analysis uses ten rounds of each method to achieve reliable comparisons.

### 5.3. Regression Scenario

In the second scenario, the proposed optimal ensemble DTACO model was compared against basic MLP regressor, SVR, and random forest regressor models over 10 runs and 80 iterations with 10 agents. In order to evaluate the effectiveness of the regression models that were applied in order to anticipate the bandwidth of the metamaterial antenna, additional measurements were used. These metrics include relative root-mean-squared error (RRMSE), Nash–Sutcliffe efficiency (NSE), mean absolute error (MAE), mean bias error (MBE), Pearson’s correlation coefficient (r), coefficient of determination (R2), and determined agreement (WI). the total number of observations in the dataset is represented by the *N* parameter. The *n*th estimated and observed bandwidth are represented by ($\hat{V_{n}}$) and ($V_{n}$), and ($\bar{\hat{V_{n}}}$) and ($V_{n}$) represents the arithmetic means of the estimated and observed values. The evaluation criteria for predictions are shown in Table 7.

The findings of the suggested optimizing ensemble DTACO-based model compared to those of the fundamental models are presented in Table 8. When compared to the RF, which had an RMSE of (0.041033), the given DTACO-based model produced the best results, with an RMSE of (0.003871). MLP, on the other hand, reported an RMSE of (0.045691), which was the poorest possible outcome.

The results of the suggested DTACO-based model’s regression are compared with the results of the DTO, ACO, WOA, GWO, GA, and PSO-based models to demonstrate the effectiveness of the presented algorithm. Table 9 provides a description of the DTACO-based model that was proposed together with the RMSE results of other models based on ten separate runs. This description includes the minimum, median, maximum, and mean average errors.

**Figure 5:**
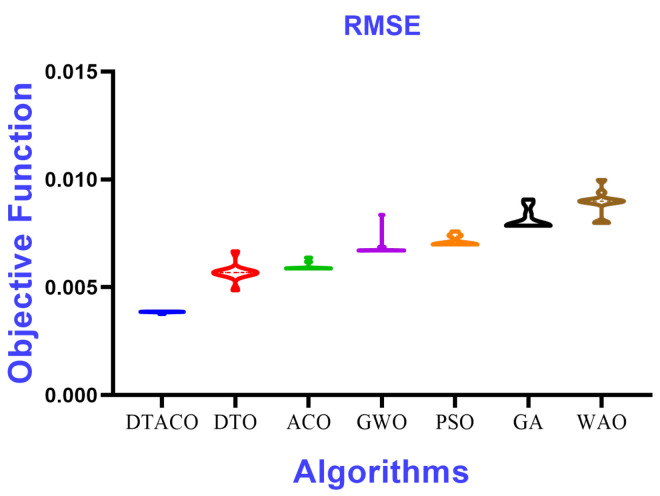
The box plot of the proposed DTACO-based model and DTO, ACO, PSO, GWO, GA, and WOA-based models based on the RMSE.

**Figure 6:**
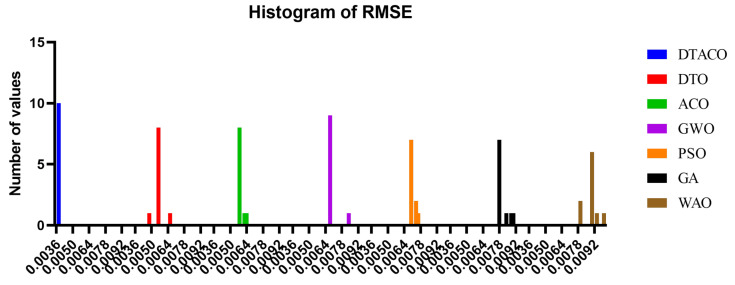
Histogram of the root-mean-squared error (RMSE) for both the DTACO-based model that was presented and the other models.

**Figure 7:**
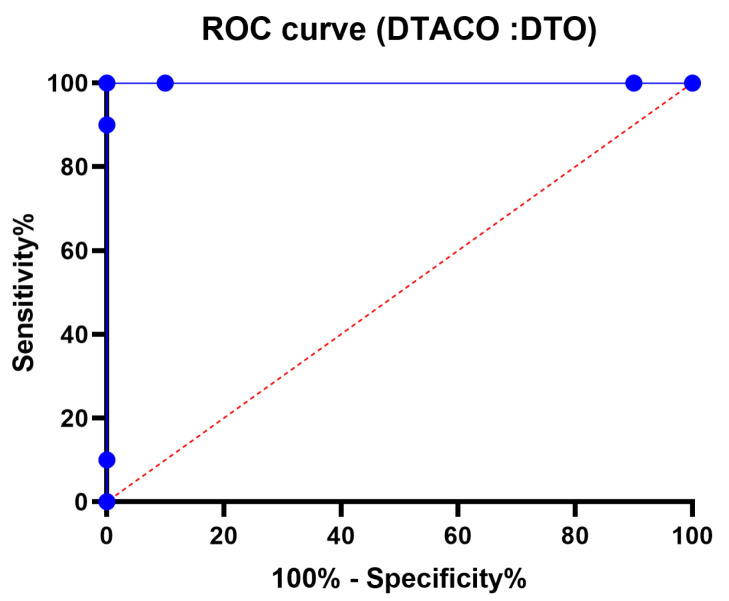
ROC curve of the presented DTACO algorithm versus the DTO algorithm.

**Figure 8:**
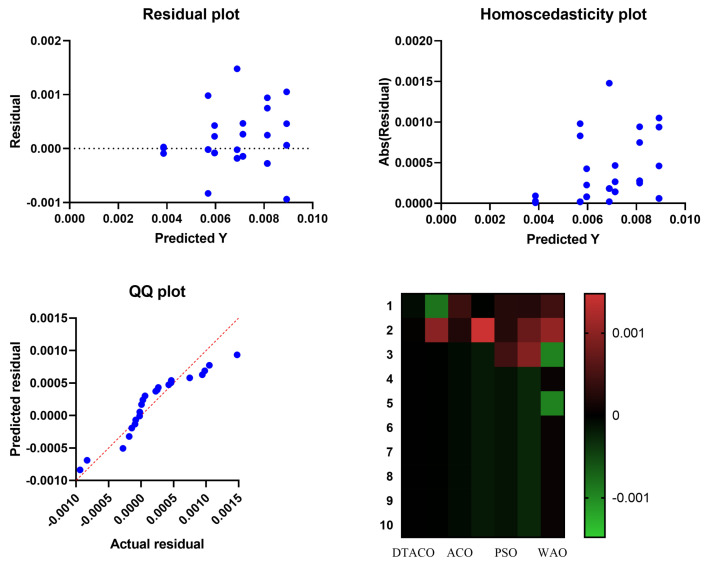
For both the models that were compared and the model that was presented using DTACO, there were QQ plots, residual plots, and heat maps.

Figure 5 displays the box plot calculated using the root-mean-squared error for the proposed DTACO-based model as well as the DTO, ACO, PSO, GWO, GA, and WOA-based models. The quality of the optimized ensemble DTACO-based model, as shown in the figure, was determined with the help of the objective function described in Equation (16). Figure 6 depicts the histogram of the root-mean-squared error (RMSE) for both the DTACO-based model that was presented and the other models. Figure 7 shows the ROC curve of the presented DTACO algorithm versus the DTO algorithm. Figure 8 presents the QQ plots, residual plots, and heat map for both the DTACO-based model that was provided and the models that were compared for the data that were investigated. Both sets of plots are based on the analyzed data. These figures demonstrate that the given optimized ensemble DTACO-based model has the potential to outperform the models that were compared.

Table 10 contains the outcomes of the ANOVA test that was performed on the proposed ensemble DTACO and the models that were compared. Table 11 contains a comparison of the proposed optimized ensemble DTACO and the models that were compared using the Wilcoxon signed-rank test. The statistical analysis was carried out by utilizing ten individual iterations of each of the algorithms that are being presented and evaluated. This ensures that the comparisons are exact and that the results of the study are reliable.

## 6. Discussion

This section summarizes the advantages and disadvantages of the suggested method when it is used in real life. The suggested method provides a better way to optimize estimating the bandwidth of a metamaterial design. It gives a methodical way to optimize the design factors of metamaterials, which leads to designs that work better and are more efficient. The method is especially useful for figuring out the bandwidth of different metamaterial designs. This is of the utmost importance in engineering applications, where it is important to make sure that developed metamaterials can work within the stated frequency range. When making metamaterials work and perform better, having an exact bandwidth prediction can be very helpful. The fact that the method has been changed to be used in engineering shows that it can be used in real-world situations and is fit for them. It is a useful tool for optimizing metamaterial design because it was made to solve problems and meet the needs of engineering uses. The described method gives designers more options for how to make things because it lets them optimize a number of factors that affect the bandwidth of metamaterials. During the optimization process, it can take into account a number of different design variables and constraints. This lets engineers look into a wide range of choices. The proposed method aims to make the optimization process more productive. By using its newly improved method, it might be possible to reduce the amount of computing time and resources needed to optimize metamaterial design. This would make it easier to use in the real world.

The suggested method focuses on improving the designs of metamaterials and predicting the bandwidth of these designs. Even though this is useful for engineering uses that use metamaterials, it may not be directly applicable to other domains or design problems because of how metamaterials work. The method will only work if accurate models and simulations of the metamaterials are being considered. Wrong or insufficient models may lead to less-than-ideal results or wrong bandwidth estimates. When different design variables and limits are considered, optimizing metamaterial designs can be difficult and time-consuming. The suggested method may still run into problems when dealing with complicated optimization problems, and may not always promise to find the global optimum. One might need the right computer resources, software tools, and experience for the suggested method to work. Engineers and researchers must consider the things mentioned here when applying the method to real-world situations.

## 7. Conclusions and Future Work

Metamaterials are unusual. They have several constituents and repeating patterns at a smaller wavelength than the phenomena they affect. Metamaterials can control electromagnetic waves by blocking, absorbing, amplifying, or bending them. Metamaterials are used in microwave invisibility cloaks, invisible submarines, revolutionary electronics, microwave components, filters, and negative-refractive-index antennas. This paper improved dipper-throated-based ant colony optimization (DTACO) to predict metamaterial antenna bandwidth. The first case examined the proposed binary DTACO algorithm’s feature selection for the dataset being reviewed, while the second scenario tested its regression. Studying both scenarios’ circumstances, DTACO was compared to the state-of-the-art DTO, ACO, PSO, GWO, and WOA algorithms. The optimal ensemble DTACO-based model was compared to the basic MLP, SVR, and random forest regressor models. The statistical research used Wilcoxon’s rank-sum and ANOVA tests to evaluate the DTACO-based model’s consistency. Because of the versatility of this method, the DTACO-based regression model can be modified and evaluated for a wide variety of datasets in work that will be performed in the future. DTACO will be evaluated with well-known benchmark functions such as CEC17-19, so that DTACO can be compared with other well-known metaheuristic algorithms in future work.

## Figures and Tables

**Table 1:** The DTACO algorithm’s configuration settings.

Parameter (s)	Value (s)
# Agents	10
# Iterations	80
# Runs	10
Dimension	# features
η	[0, 1]
$\eta^{\prime }$	[0, 1]
Mutation probability	0.5
Exploration percentage	70
Pheromone evaporation factor (ρ)	0.1
Pheromone factor (α)	1
Heuristic factor (β)	1
Intensity value (*Q*)	0.2
$h_{1}$ of $f_{n}$	0.99
$h_{2}$ of $f_{n}$	0.01

**Table 2:** Compared algorithms’ various configuration parameters.

Algorithm	Parameter (s)	Value (s)
DTO	η	[0, 1]
	$\eta^{\prime }$	[0, 1]
	Mutation probability	0.5
	Exploration percentage	70
	Birds	10
	Iterations	80
ACO	Pheromone evaporation factor (ρ)	0.1
	Pheromone factor (α)	1
	Heuristic factor (β)	1
	Intensity value (*Q*)	0.2
	Ants	10
	Iterations	80
GWO	*a*	2 to 0
	Wolves	10
	Iterations	80
PSO	Acceleration constants	[2, 2]
	Inertia $W_{min}$, $W_{max}$	[0.6, 0.9]
	Particles	10
	Iterations	80
GA	Cross over	0.9
	Mutation ratio	0.1
	Selection mechanism	Roulette wheel
	Agents	10
	Iterations	80
WOA	*r*	[0, 1]
	*a*	2 to 0
	Whales	10
	Iterations	80

**Table 3:** Feature selection evaluation criteria.

Metric	Formula
Best fitness	$min_{i=1}^{M}S_{i}^{*}$
Worst fitness	$max_{i=1}^{M}S_{i}^{*}$
Average error	$\frac{1}{M}\sum_{j=1}^{M}\frac{1}{N}\sum_{i=1}^{N}mse\left(\hat{V_{i}}-V_{i}\right)$
Average fitness	$\frac{1}{M}\sum_{i=1}^{M}S_{i}^{*}$
Average fitness size	$\frac{1}{M}\sum_{i=1}^{M}size\left(S_{i}^{*}\right)$
Standard deviation	$\sqrt{\frac{1}{M-1}\sum_{i=1}^{M}{\left(S_{i}^{*}-Mean\right)}^{2}}$

**Table 4:** Proposed binary DTACO versus other optimization algorithms.

	bDTACO	bDTO	bACO	bPSO	bGWO	bGA	bWOA
Average error	**0.5027**	0.5265	0.5308	0.5472	0.571	0.5694	0.5708
Average Select size	**0.4728**	0.8061	0.6152	0.6728	0.6728	0.7073	0.8362
Average Fitness	**0.5832**	0.6077	0.6108	0.5994	0.5978	0.6497	0.6056
Best Fitness	**0.485**	0.5612	0.5141	0.5197	0.5781	0.5684	0.5697
Worst Fitness	0.5835	**0.6712**	0.6292	0.5866	0.6458	0.666	0.6458
Standard deviation Fitness	**0.4055**	0.4284	0.4118	0.4102	0.4096	0.4464	0.4118

**Table 5:** The outcomes of the ANOVA test for the suggested algorithm and the algorithms under comparison.

	SS	DF	MS	F (DFn, DFd)	*p* Value
Treatment (between columns)	0.04667	6	0.007779	F (6, 63) = 311.2	*p* < **0.0001**
Residual (within columns)	0.001575	63	0.000025	-	-
Total	0.04825	69	-	-	-

**Table 6:** Wilcoxon signed-rank test results of the proposed bDTACO and other optimization algorithms.

	bDTACO	bDTO	bACO	bPSO	bGWO	bGA	bWOA
Theoretical median	0	0	0	0	0	0	0
Actual median	**0.5027**	0.5265	0.5308	0.5472	0.571	0.5694	0.5708
Number of values	10	10	10	10	10	10	10
Wilcoxon signed-rank test							
Sum of signed ranks (W)	55	55	55	55	55	55	55
Sum of positive ranks	55	55	55	55	55	55	55
Sum of negative ranks	0	0	0	0	0	0	0
*p* value (two-tailed)	**0.002**	**0.002**	**0.002**	**0.002**	**0.002**	**0.002**	**0.002**
Exact or estimate?	Exact	Exact	Exact	Exact	Exact	Exact	Exact
Significant (alpha = 0.05)?	Yes	Yes	Yes	Yes	Yes	Yes	Yes
How big is the discrepancy?							
Discrepancy	**0.5027**	0.5265	0.5308	0.5472	0.571	0.5694	0.5708

**Table 7:** Evaluation criteria for predictions.

Metric	Formula
RMSE	$\sqrt{\frac{1}{N}\sum_{n=1}^{N}{\left(\hat{V_{n}}-V_{n}\right)}^{2}}$
RRMSE	$\frac{RMSE}{\sum_{n=1}^{N}\hat{V_{n}}}\times 100$
MAE	$\frac{1}{N}\sum_{n=1}^{N}\left|\hat{V_{n}}-V_{n}\right|$
MBE	$\frac{1}{N}\sum_{n=1}^{N}\left(\hat{V_{n}}-V_{n}\right)$
NSE	$1-\frac{\sum_{n=1}^{N}{\left(V_{n}-\hat{V_{n}}\right)}^{2}}{\sum_{n=1}^{N}{\left(V_{n}-\bar{\hat{V_{n}}}\right)}^{2}}$
WI	$1-\frac{\sum_{n=1}^{N}\left|\hat{V_{n}}-V_{n}\right|}{\sum_{n=1}^{N}|V_{n}-\bar{V_{n}}\left|+\right|\hat{V_{n}}-\bar{\hat{V_{n}}}|}$
R2	$1-\frac{\sum_{n=1}^{N}{\left(V_{n}-\hat{V_{n}}\right)}^{2}}{\sum_{n=1}^{N}{\left(\sum_{n=1}^{N}V_{n})-V_{n}\right)}^{2}}$
r	$\frac{\sum_{n=1}^{N}\left(\hat{V_{n}}-\bar{\hat{V_{n}}}\right)\left(V_{n}-\bar{V_{n}}\right)}{\sqrt{\left(\sum_{n=1}^{N}{\left(\hat{V_{n}}-\bar{\hat{V_{n}}}\right)}^{2}\right)\left(\sum_{n=1}^{N}{\left(V_{n}-\bar{V_{n}}\right)}^{2}\right)}}$

**Table 8:** Proposed optimizing ensemble DTACO model versus basic models’ results.

	RMSE	MAE	MBE	r	R2	RRMSE	NSE	WI
MLP	0.045691	0.034697	0.003041	0.979418	0.959260	14.020177	0.958770	0.911594
SVR	0.042958	0.033554	0.005764	0.981950	0.964225	13.181572	0.963555	0.914505
RF	0.041033	0.029360	−0.001995	0.983923	0.968104	29.856423	0.966747	0.925193
Ensemble DTACO	**0.003871**	**0.006723**	**−0.000237**	**0.999048**	**0.998096**	**3.028931**	**0.998076**	**0.982871**

**Table 9:** Description of the proposed DTACO-based model and other models’ results from RMSE.

	bDTACO	bDTO	bACO	bPSO	bGWO	bGA	bWOA
Number of values	10	10	10	10	10	10	10
Minimum	**0.00377**	0.004868	0.00588	0.00671	0.00699	0.00786	0.00799
Maximum	**0.00389**	0.00668	0.006388	0.008371	0.007599	0.009079	0.00998
Range	**0.00012**	0.001812	0.000508	0.001661	0.000609	0.001219	0.00199
Mean	**0.003862**	0.005699	0.005962	0.006892	0.007133	0.008137	0.008929
Std. deviation	**3.29 $\times \;10^{-5}$**	0.000429	0.000178	0.000522	0.000236	0.000477	0.000587
Std. error of Mean	**1.04** $\times \;10^{-5}$	0.000136	5.64 $\times \;10^{-5}$	0.000165	7.47 $\times \;10^{-5}$	0.000151	0.000186
Harmonic mean	**0.003862**	0.00567	0.005957	0.006862	0.007126	0.008114	0.008893
Skewness	−2.927	**0.6532**	2.075	3.11	1.253	1.436	−0.2714
Kurtosis	9.076	4.709	3.431	9.739	**−0.1102**	0.4966	0.8392

**Table 10:** The outcomes of the ANOVA test for the comparison models and the suggested ensemble DTACO.

	SS	DF	MS	F (DFn, DFd)	*p* Value
Treatment (between columns)	0.000169	6	0.00002808	F (6, 63) = 175.9	*p* < **0.0001**
Residual (within columns)	1.01 $\times \;10^{-5}$	63	1.596 $\times \;10^{-7}$	-	-
Total	0.000179	69	-	-	-

**Table 11:** Comparison between the models that were compared using the Wilcoxon signed-rank test and the proposed ensemble DTACO.

	bDTACO	bDTO	bACO	bPSO	bGWO	bGA	bWOA
Theoretical median	0	0	0	0	0	0	0
Actual median	**0.00387**	0.00568	0.00588	0.00671	0.00699	0.00786	0.00899
Number of values	10	10	10	10	10	10	10
Wilcoxon signed-rank test							
Sum of signed ranks (W)	55	55	55	55	55	55	55
Sum of positive ranks	55	55	55	55	55	55	55
Sum of negative ranks	0	0	0	0	0	0	0
*p* value (two-tailed)	**0.002**	**0.002**	**0.002**	**0.002**	**0.002**	**0.002**	**0.002**
Exact or estimate?	Exact	Exact	Exact	Exact	Exact	Exact	Exact
Significant (alpha = 0.05)?	Yes	Yes	Yes	Yes	Yes	Yes	Yes
How big is the discrepancy?							
Discrepancy	**0.00387**	0.00568	0.00588	0.00671	0.00699	0.00786	0.00899

## Data Availability

Not applicable.

## Abbreviations

kNN	k-Nearest Neighbors
MLP	Multilayer Perceptron
SVR	Support Vector Regression
RF	Random Forest
DTO	Dipper-Throated Optimization
ACO	Ant Colony Optimization
PSO	Particle Swarm Optimization
WOA	Whale Optimization Algorithm
GWO	Grey Wolf Optimizer
